# Ewha leading the era of great transformation through inclusive innovation for a sustainable future: a presidential inaugural address

**DOI:** 10.12771/emj.2025.00108

**Published:** 2025-03-25

**Authors:** Hyang-Sook Lee

**Affiliations:** The 18th President, Ewha Womans University, Seoul, Korea

It is an honor to stand before you today as the 18th president of Ewha Womans University (Fig. 1).

I extend a warm welcome and sincere gratitude to Chairperson Myong-sue Chang and the esteemed members of the Board of Trustees; to former presidents of Ewha—including my immediate predecessor, President Eun Mee Kim; to President Myung Kyung Lee of the Ewha Alumnae Association; and to University Chaplain Reverend Sunhee Ahn; as well as to the many distinguished guests who have honored this occasion with their presence despite their busy schedules. I also express my deepest appreciation to our faculty, staff, students, and alumnae, with whom we are building a vibrant Ewha community.

## Ewha’s proud history

Today, carrying forward Ewha’s proud history and tradition, I stand before you to confront the challenges of a new era, establish a vision for our future, and chart a blueprint for its realization—all inspired by the remarkable achievements of our former presidents and alumnae. Founded in 1886 through the prayers and dedication of missionary Mary F. Scranton, the first principal of Ewha Haktang, our institution was established in the spirit of love and service to the Lord. I am profoundly grateful for God’s grace and dedicate all glory to Him for granting me the opportunity to continue this sacred mission as the 18th president of Ewha. I sincerely thank the Ewha family for their steadfast faith and for entrusting me with this vital responsibility. I pledge to be a president who fulfills God’s vision and benevolent purpose for Ewha.

Ewha’s history is a testament to God’s providence, and our journey has been one of faithfully serving His Word. From humble beginnings with a single student to the extraordinary accomplishment of nurturing 260,000 distinguished alumnae, Ewha’s story is not merely that of an educational institution but a journey of faith that has empowered women in this country to discover their true selves and dedicate their lives to serving the world. This year is especially significant as it marks both the 100th anniversary of Ewha College’s foundation in 1925 and the 90th anniversary of its relocation from Jeong-dong to Sinchon in March 1935—a move that established a new learning environment.

In 1946, sixty years after laying the cornerstone for women’s education, Ewha Haktang was officially accredited as Korea’s first 4-year university. Another 50 years later, in 1996, Ewha made history again by establishing the world’s first engineering college for women, thus opening a new chapter in nurturing engineering talent. Throughout its history, Ewha has achieved numerous “firsts” and “bests,” solidifying its reputation as a pioneer ahead of its time. Ewha’s journey has always signified the first step toward a new era—a tradition that endures to this day.

## Ewha’s role in women’s education and society innovation

Ewha’s endeavors extend well beyond women’s education; they have become a driving force behind the transformative changes that have shaped modern Korean society. Throughout the 20th century, Ewha was recognized not only as an institution of higher learning but also as a symbol of boldness and innovation that propelled the nation’s remarkable development and globalization. There was never any doubt that Ewha’s path provided a righteous direction to guide Korean society into the future.

Today, amid a vast current of change driven by cutting-edge technologies, such as artificial intelligence (AI), we face fundamental questions that call for a redefinition of the role and status of universities. Simultaneously, climate-induced environmental changes, a declining school-age population, and growing political and economic instability intensify our concerns about the future direction of higher education. In the face of these internal and external challenges, it falls upon Ewha to respond wisely and proactively, leading the way in addressing current issues and actively shaping the future.

As a member and president of Ewha—an institution that has demonstrated resilience by achieving extraordinary outcomes and significant progress in times of crisis—I am confident that our collective wisdom and capabilities will enable us to overcome the challenges ahead. To this end, I wish to emphasize the spirit of “Ilsinuilsin (日新又日新),” meaning “to renew and improve oneself day by day.” This guiding principle, rooted in the historical legacy of Ewha College and its leadership in women’s education, is essential for fostering the academic creativity required in this era of technological transformation.

Beginning with education in the humanities, social sciences, and arts—including literature, music, and early childhood education—Ewha has consistently innovated to meet the demands of the times. Even during the challenging period following Korea’s liberation, we laid strong academic foundations in diverse fields, including the natural sciences. At the heart of these advancements is Ewha’s unique spirit of creating new values and pursuing reinvention, anchored in a Christian ethos of serving the world with righteous influence and discipline. Despite an uncertain future, Ewha has persistently charted a virtuous course and built a forward-looking, sustainable model for universities—a mission that defines our role as a global institution today.

## Ewha’s 6 visions leading the era of great transformation

Today, at this inauguration ceremony, I am proud to declare a new vision inspired by the zeitgeist: “Ewha Leading the Era of Great Transformation through Inclusive Innovation.” Through creative and inclusive innovation, Ewha will establish people-centered values and spearhead change in this era of rapid technological transformation. Moreover, we will continually renew and improve ourselves to benefit society and lead our times by upholding the core values of creativity and challenge, excellence and innovation, cooperation and companionship, sustainability, and sharing and service. To instill pride among all members of our community and to elevate our capabilities and competitiveness, I will implement the following policies:

First, in research, I will create a world-class environment to bolster competitiveness through first-mover research leadership. I will cultivate an optimal setting that supports focused research and enables our faculty to achieve world-class outcomes. Additionally, I will recruit top-tier faculty by introducing flexible contracts, performance-based rewards, and securing special-purpose funds. I will work diligently to establish a virtuous cycle in which Ewha’s technological innovations benefit society while further enhancing our research capacity through technology commercialization. I will also promote transdisciplinary convergence research that spans Ewha’s diverse academic fields, ensuring balanced development between fundamental and applied disciplines and expanding research opportunities for the next generation of scholars.

Second, in education, I aim to establish Ewha’s model for future learning, positioning us to lead the transformation of higher education in the era of AI. Advances in AI represent more than mere technological innovation; they fundamentally alter our lives and thought processes, offering new experiences and possibilities. In this era of change, Ewha is committed to nurturing global women leaders who contribute to national and human advancement, guided by an educational philosophy rooted in our founding Christian values of truth, goodness, and beauty. Building on our outstanding educational system and infrastructure, I will establish an AI-based framework that includes the “AI for All Ewha” initiative and customized AI programs for students across various majors. I will support our students in developing digital capacities—including problem-setting and problem-solving, creative thinking, innovation, as well as collaboration and communication—while enhancing their AI literacy and expertise. I will provide an educational environment and programs optimized to help our students cultivate unique talents in the AI era.

Third, regarding administrative infrastructure, I will maximize Ewha’s growth potential by innovating our administrative systems and advancing campus infrastructure. I will implement an administrative framework grounded in accountability and trust by enhancing staff professionalism and introducing diligent human resources and accountability practices. Additionally, I will establish a foundation for sustainable growth by expanding the systems required for creating an optimal environment for focused research and educational excellence. In particular, I will faithfully execute the campus master plan, including the construction of the EWC, in preparation for Ewha’s future.

Fourth, concerning our internal and external environments, I will make bold and strategic efforts to enhance Ewha’s brand value and global standing. Drawing on the distinctive values of our time-honored history and tradition, Ewha will further solidify its position as a world-class research and education institution through academic excellence, social responsibility, and global solidarity. In doing so, I will uphold the virtues befitting a prestigious global private university and continue to expand Ewha’s positive influence in the international community.

Fifth, a sustainable financial expansion system is essential for university development. For Ewha to continue growing, financial security must be a top priority. I will significantly expand our finances by securing funding through the Ewha University-Industry Collaboration Foundation, boosting fundraising by strengthening external cooperation structures, increasing educational projects, advancing fund management practices, capitalizing on Ewha’s brand value, and developing profit-generating businesses.

Sixth, the Ewha University Medical Center (EUMC) is a proud pillar of Korean history alongside Ewha itself. Launched in 1887 as Po Goo Nyo Goan (普救女館), Korea’s first exclusive hospital for women, the EUMC has pioneered the education of female medical professionals in Korea by producing the country’s first female doctors and nurses, as well as providing medical treatment to the underprivileged. Based on this long history and tradition, Ewha, along with EUMC Mokdong, EUMC Seoul, and its College of Medicine, will create synergy through cooperation for shared growth, strengthen research and treatment capabilities by establishing an Ewha cutting-edge convergence med-healthcare cluster, and enhance the global competitiveness of the EUMC.

**Fig. 2.**
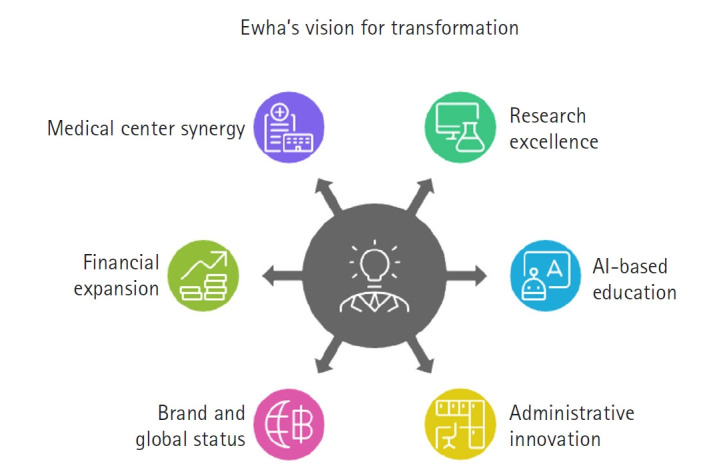
Diagram of Ewha’s 6-fold vision for leading the era of great transformation.

Ewha’s 6-fold vision is illustrated in Fig. 2.

## Humble request for active participation and cooperation

To pursue these goals and policies, the active participation and cooperation of all Ewha members is essential.

Dear Ewha students,

I am keenly aware of your concerns about an uncertain future. However, as you come to recognize that your reverence for the work of the Lord—the true founder of Ewha—is the genuine source of wisdom in embracing challenges, you will soon evolve into creative and enterprising global female leaders, equipped with both Christian values and intellect. I hope that you will nurture your aspirations to become true leaders who contribute to the global community as citizens of the world. I am committed to ensuring that Ewha remains a place where you can freely pursue your dreams and overcome every challenge, fostering a spirit of boundless ambition within our community.

Dear Ewha Womans University staff,

You have devoted yourselves to the growth and development of Ewha, serving as the steadfast pillars of our institution and as the primary agents of change and innovation on campus. The continued progress and advancement of Ewha depend on your effort, passion, and expertise. I kindly ask you to align with Ewha’s vision and goals, as you have proudly done in the past, and I trust that you will continue collaborating in support of our mission. Please help create an inspiring and joyful workplace founded on respect, collaboration, and fairness. I, too, will commit my utmost to this shared journey.

Dear esteemed Ewha faculty members,

I express my deepest gratitude and respect for your unwavering dedication to education and research—Ewha’s excellence is a direct result of your academic achievements and continuous efforts. Amid a rapidly changing environment, the university requires a new vision and innovative endeavors. I will spare no effort to support those of you who remain steadfast during these challenging times, both internally and externally, so that together we may achieve the highest possible outcomes. I invite you to join us on this journey as Ewha takes a great leap forward as a prestigious global private university, embracing inclusive innovation in the face of new challenges.

Dear proud Ewha alumnae,

Ewha’s reputation and tradition are built on your dedicated efforts and outstanding achievements. Our alumnae, who demonstrate leadership across all fields and embody the spirit of Ewha, are a source of immense pride and a cherished asset to our institution. Your ongoing support and encouragement have always been a strong foundation of our success. As Ewha advances toward a new leap forward, I ask for your unwavering encouragement, continued interest, and steadfast support for the development of your alma mater and the growth of current Ewha students. In return, Ewha will strive to excel—even in times of great transformation—and remain an alma mater of which you can be proud.

Dear distinguished guests and Ewha family members,

Throughout history and across cultures, universities have consistently been at the forefront of driving national competitiveness. Ewha is committed to fulfilling its role as a leading institution in this era of immense challenges and uncertainty. We will meet society’s demands by nurturing creative, future-ready talent for the digital age. Through world-class research, development, and innovation—the cornerstones of national competitiveness—Ewha will serve as a powerful engine for growth. Building on 139 years of unwavering dedication to its social responsibilities, Ewha will continue to thrive as a sustainable social asset for future generations.

## Character before knowledge and love before technology

Under the vision of “Ewha leading the era of great transformation through inclusive innovation,” we strive to advance as a new university worthy of the 21st century. As a prestigious global private university that values individual dignity and character, Ewha is preparing for a new era of science and technology. By aligning research projects with our faculty, financial resources with the institution, and our prestige with the global community—while upholding the core values of creativity and challenge, excellence and innovation, cooperation and companionship, sustainability, and sharing and service—we aim to forge a new Ewha as we embark on a journey toward the future.

Reflecting on the sage words of President Kim Okgill, “Ewha is a house of learning that teaches character before knowledge and love before technology,” I will devote my heart and sincerity to guiding Ewha’s next leap forward. To this end, I maintain a diary called the “Ewha Notes,” in which I document my reflections from meetings with various members of our community and their heartfelt insights about our institution. Cherishing the “Ewha Notes” as a moral compass, I pledge to do my utmost to establish Ewha as a prestigious private university renowned worldwide. From this moment on, I vow to undertake this bold journey together with all members of the Ewha community.

“Trust in the Lord with all your heart, and lean not on your own understanding. In all your ways acknowledge Him, and He will make your paths straight,” Proverbs 3:5-6. As we walk with the Lord, guided by His love for Ewha and its members and humbly seeking His wisdom and understanding, He will illuminate the righteous path for our future.

Dear distinguished guests and esteemed Ewha family members, I pray that the Lord’s endless grace and blessings be with you, and that His righteousness and love flow abundantly through Ewha.

Thank you.

## Figures and Tables

**Fig. 1.**
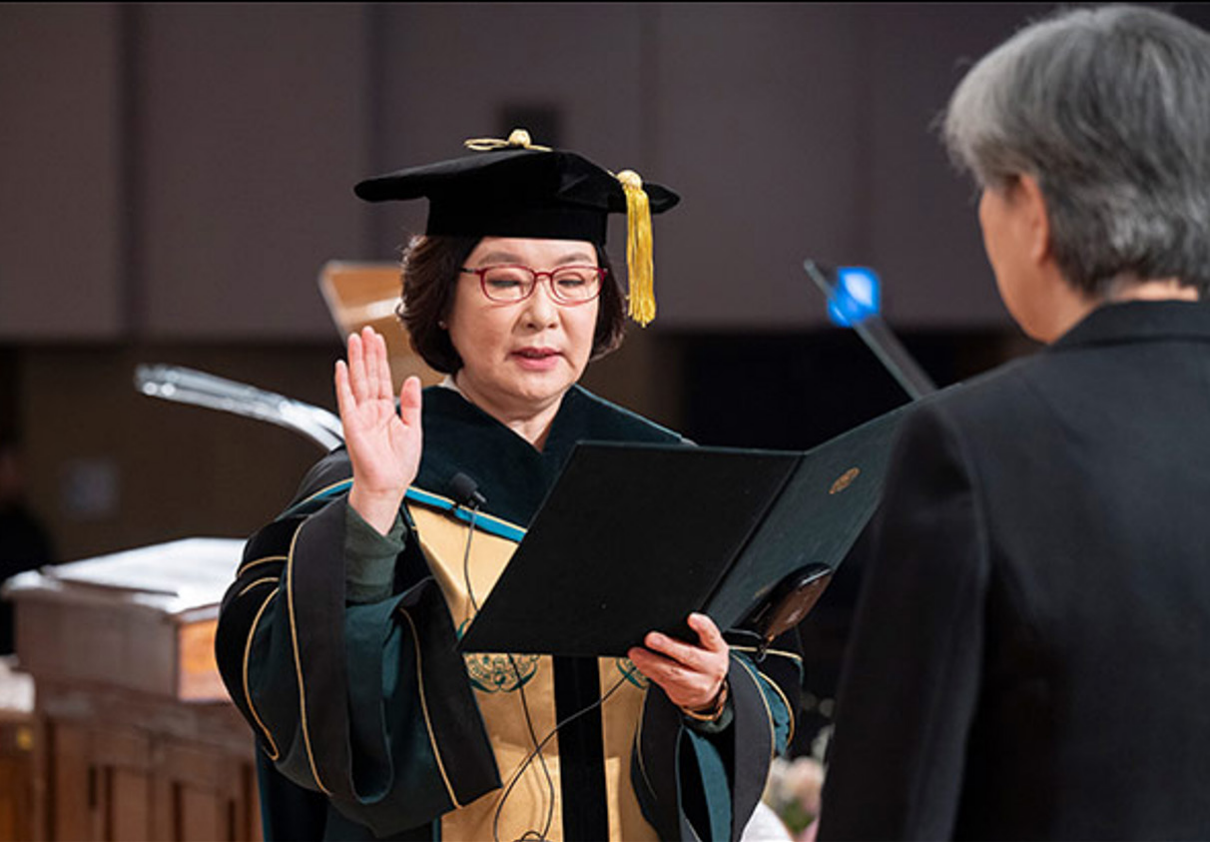
Hyang-Sook Lee. The 18th president of Ewha Womans University.

